# A Physic Nut Stress-Responsive HD-Zip Transcription Factor, *JcHDZ07*, Confers Enhanced Sensitivity to Salinity Stress in Transgenic *Arabidopsis*

**DOI:** 10.3389/fpls.2019.00942

**Published:** 2019-07-17

**Authors:** Yuehui Tang, Xinxin Bao, Shuang Wang, Yan Liu, Jie Tan, Mengxia Yang, Mengyuan Zhang, Rongrong Dai, Xinrong Yu

**Affiliations:** ^1^Key Laboratory of Plant Genetics and Molecular Breeding, Zhoukou Normal University, Zhoukou, China; ^2^Henan Key Laboratory of Crop Molecular Breeding and Bioreactor, Zhoukou Normal University, Zhoukou, China; ^3^School of Journalism and Communication, Zhoukou Normal University, Zhoukou, China

**Keywords:** physic nut, HD-Zip transcription factor, *JcHDZ07*, salinity stress, transgenic *Arabidopsis*

## Abstract

Homeodomain-leucine zipper (HD-Zip) transcription factors are reported to play crucial roles in the growth, development, and stress responses of plants. However, there is little knowledge of the molecular mechanisms involved in physic nut’s stress tolerance generally, or the functions of its *HD-Zip* genes. In the present study, a HD-Zip family transcription factor, designated *JcHDZ07*, was isolated from physic nut. Expression profile analysis showed that salinity stress inhibited the expression of *JcHDZ07*. Transient expression of JcHDZ07-YFP in *Arabidopsis* protoplast cells revealed that JcHDZ07 was a nuclear-localized protein. Additionally, no obvious difference in growth and development between wild-type and *JcHDZ07*-overexpressing plants was observed in the absence of stress. Our results further indicated that *JcHDZ07* overexpressing transgenic plants had lower proline contents, lower survival rates, and activities of catalase and superoxide dismutase, but higher relative electrical leakage and malonaldehyde contents compared with wild-type plants under salinity stress conditions, suggesting that overexpression of *JcHDZ07* confers enhanced sensitivity to salinity stress in transgenic *Arabidopsis*. Expression of salt stress-responsive genes were upregulated in leaves of transgenic plants under salinity stress, but less strongly than in wild-type plants. Collectively, our results suggest that *JcHDZ07* functions as an important regulator during the process of plant responses to salinity stress.

## Introduction

Salinity is a major inhibitor of plant growth and development that severely impairs crop yields in many regions. To cope with salinity stress, plants have evolved diverse physiological and biochemical strategies to adapt to unfavorable environmental conditions, which include accumulating proline and protective proteins, reducing MDA, and increasing the level of antioxidants (Parida and Das, 2005; Sah et al., 2016). Such cascading events are controlled by a series of stress-responsive genes through complex regulatory networks (Sah et al., 2016). In these regulating processes, transcription of these genes are largely controlled by specific transcription factors. Several studies have clearly demonstrated that numerous transcription factors, belonging to MYB, AP2/ERF, HD-Zip, WRKY, and NAC families, have been identified, characterized and shown to participate in regulation of plants’ responses to abiotic stresses (Mizoi et al., 2012; Shao et al., 2015; Perotti et al., 2017; Erpen et al., 2018). Transgenic plants overexpressing these groups of genes have suggested improved resistance to various stresses (Erpen et al., 2018).

HD-Zip proteins constitute a large plant-specific family of transcription factors. The HD-Zip proteins are characterized by a highly conserved DNA-binding homeodomain (HD) and a leucine zipper (Zip) motif that mediates homo- and hetero-dimerization (Henriksson et al., 2005). Based on the DNA-binding specificity, sequence similarities and the presence of additional domains, 48 *Arabidopsis* HD-Zip proteins are classified into four subfamilies (HD-Zip I–IV) (Sessa et al., 1994).

Since the first isolation of an HD-containing gene, *KNOTTED1* from maize, numerous HD-Zip transcription factors have been identified from various plant species (Turchi et al., 2015). Further studies indicate that HD-Zip proteins play essential roles in diverse aspects of plant growth and development (Perotti et al., 2017; Roodbarkelari and Groot, 2017). For example, *ATHB12* acts as a positive regulator of cell growth during leaf development (Hur et al., 2015), and overexpression of *ATHB-8* promotes vascular cell differentiation in *Arabidopsis* (Baima et al., 2001). It has been reported that progressive loss of *HAT3, ATHB4*, and *ATHB2* activity in *Arabidopsis* causes developmental defects in embryogenesis (Turchi et al., 2013). *PtrHB7* plays a critical role in regulation of vascular cambium differentiation in populous in a dosage-dependent manner (Zhu et al., 2013). *PtrHB4* is required for interfascicular cambium formation to develop the vascular cambium in woody species (Zhu et al., 2018). In rice, *OsHox33* knockdown accelerates leaf senescence by regulating expression of *GS1* and *GS2* (Luan et al., 2013). *OsHox32* is reportedly involved in leaf development, and transgenic plants overexpressing it produce narrow leaves (Li et al., 2016). In addition to the functions described above, HD-Zip proteins are also participate in response to various abiotic stresses. For example, *MtHB2* has been shown to play a role in the adaptive response to drought and salinity stresses in *Arabidopsis* (Song et al., 2011). Over-expression of *OsHOX24* increases transgenic plants sensitivity to salinity stress as compared to WT plants (Bhattacharjee et al., 2016). Overexpression of *Zmhdz10* improves drought and salinity tolerance in rice (Zhao et al., 2014). Taken together, although members of the HD-Zip family have been extensively cloned and functionally studied, these genes remain relatively poorly characterized in perennial species, especially members of the physic nut.

Physic nut is one of the most promising energy plants for tropical and sub-tropical regions (Openshaw, 2000). Physic nut is being widely cultivated, partly because of its high drought and salinity tolerance (Openshaw, 2000), however, the mechanisms underlying its salinity stress tolerance remain unclear. In previous report, we notice that a HD-Zip transcription factor, we named *JcHDZ07*, which is strongly repressed expression by salinity stress (Zhang et al., 2014). Therefore, *JcHDZ07* gene was chosen for further analysis. In the present study, we analyzed the expression profile of *JcHDZ07* gene under non-stressed and exposed to drought and salt stresses conditions, and functionally characterized the role of *JcHDZ07* in response to salinity stress by overexpressing *JcHDZ07* in *Arabidopsis*. Our findings show that overexpressing *JcHDZ07* in *Arabidopsis* rendered the transgenic lines more sensitivity to salinity stress. The research will provide a useful foundation for further research into molecular mechanisms underlying stress responses in physic nut, and plants generally.

## Materials and Methods

### Plant Materials

The inbred cultivar GZQX0401 of physic nut was used for our research. Physic nut seedlings were grown in pots containing mixed soil (soil was mixed with sand at 1:3 ratio) in a greenhouse with a cycle of 16 h light/8 h dark at 28 ± 1°C. In addition, seedlings were irrigated daily with Hoagland solution. Roots, stem cortex, and leaves of 3-week-old seedlings, flowers, and seeds at 25 days after pollination, were used to analyze the organ-specific expression patterns of *JcHDZ07* in physic nut. For salinity stress, Hoagland solution containing 150 mM NaCl was used to irrigate six-leaf seedlings. For drought stress, Hoagland solution containing 20% PEG6000 was used to irrigate six-leaf seedlings. The fourth leaves of 0, 1, 3, 6, and 12 h after salinity and drought stresses were collected, and then were stored at -80°C for subsequent analysis.

*Arabidopsis thaliana* (Columbia ecotype) was used as wild-type and transgenic analysis. *Arabidopsis* seedlings were grown in a growth room at 22 ± 2°C under 16/8 h (light/dark) conditions.

### Protein Sequence and Phylogenetic Analyses

HD-Zip amino acid sequences of *Arabidopsis* from the TAIR^1^ database. HD-Zip proteins of physic nut were downloaded from the NCBI^2^ websites. MEGA 6 was selected to construct a Neighbor-Joining tree by bootstrapping with the following parameters: 1,000 bootstrap replications, Poisson model, and treatment of gaps/missing data as complete deletions.

### Subcellular Localization of JcHDZ07 Protein

The open reading frame (ORF) sequence of the *JcHDZ07* gene without the stop codon was amplified by RT-PCR using using cDNA generated from RNA extracted from physic nut root and leaf samples as a template. After confirmatory DNA sequencing, the sequence of *JcHDZ07* was fused to the 5’-terminal end of the YFP gene and put under the control of the cauliflower mosaic virus (CaMV) 35S promoter in the pSAT6-eYFP-N1 vector. The pSAT6-eYFP-N1 vector (35S::YFP) and the pSAT6-JcHDZ07-eYFP construct (35S::JcHDZ07-YFP) were transformed into *Arabidopsis* protoplasts using the PEG (polyethylene glycol) mediated method. *Arabidopsis* protoplasts were prepared following Abel and Theologis (2010). YFP fluorescence signals were excited at 514 nm and detected under a Zeiss LSM 510 Meta confocal laser scanning microscope (Oberkochen, Germany) using a 530–580 nm emission filter. Chlorophyll autofluorescence signals were excited at 552 nm and detected under a Zeiss LSM 510 Meta confocal laser scanning microscope (Oberkochen, Germany) using a 650–680 nm emission filter.

### Cloning of *JcHDZ07* and Plant Transformation

The full-length coding sequences of *JcHDZ07* was amplified with the primer pairs shown in Supplementary Table S1 by RT-PCR with 2 μL cDNA from roots and leaves. The PCR reaction was carried out with PrimeSTAR^®^ HS DNA Polymerase (TaKaRa, Beijing, China) in a total volume of 20 μL with an initial denaturing step at 98°C for 3 min, 32 cycles of 10 s at 98°C, 5 s at 54°C, and 70 s at 72°C, and a final extension step of 7 min at 72°C. The amplification products were cloned into the pMD18-T vector (TaKaRa, Beijing, China), and then sequencing. The target sequence was cloned into the *Kpn* I–*Xba I* sites of the pCAMBIA1301 vector under the control of the CaMV 35S promoter. The resulting construct was introduced into *Agrobacterium tumefaciens* (strain GV3101) by the freeze-thaw procedure. Finally, *Agrobacterium* harboring the constructs was transferred into *Arabidopsis* plants by the floral dip transformation method (Clough and Bent, 1998). The transgenic plants were screened on 1/2 MS medium with 25 mg L^-1^ hygromycin. Subsequently, semi-quantitative RT-PCR was selected to analyze the expression of *JcHDZ07* in transgenic *Arabidopsis* plants. Homozygous transgenic plants of the T3 generation were used for subsequent experiments.

### Phenotype Analysis of Transgenic Plants With *JcHDZ07*

Seeds of transgenic and wild-type plants were surface-sterilized and sown on the 1/2 MS medium and cultured vertically. After 4 days, wild-type and transgenic plant seedlings with consistent growth were selected and transferred to new vertical 1/2 MS medium and 14 cm deep circular plates filled with a 1:3 mixture of nutrient soil and vermiculite for growth at 22 ± 2°C under 16/8 h (light/dark) conditions in a growth chamber. After 7 days, the morphology of seedling roots grown in 1/2 MS medium was observed, and the length of the main roots was counted. After 14 days, the phenotypes of *Arabidopsis* seedlings grown in nutrient soil and vermiculite were observed. Furthermore, a total of 45 individual plants each of the transgenic (OE1, OE2, and OE3) and wild-type plants were used to analyze the flowering time, 1,000-seed weight and yield per plant.

### Stress Treatments

Seeds of *JcHDZ07*-overexpressing and wild-type *Arabidopsis* plants were surface-sterilized, and then put the seeds in 1.5 mL EP tube containing sterile water for 2 days in the dark at 4°C. For salinity stress, after 2 days, seeds of wild-type and transgenic plants were spotted into 1/2 MS medium containing 0 and 100 mM NaCl. Then culture vessels containing the seeds were incubated at 22 ± 2°C under 16/8 h (light/dark) conditions in a growth chamber. After 14 days, leaves were collected and used for detecting the relative electrolyte leakage (REL), Malondialdehyde (MDA) and proline content, and superoxide dismutase (SOD) and catalase (CAT) activities. After 20 days, the phenotype of wild-type and transgenic plants was observed, and the survival rates were calculated. Similar results were obtained with three biological replicates. For drought stress, after 4 days, wild-type and transgenic plant seedlings with consistent growth were selected and transferred to new vertical 1/2 MS medium containing 0 and 20% PEG6000. After 7 days, the morphology of seedlings was observed. The experiment contained three biological replicates. For salinity stress, in adult *Arabidopsis*, 10-week-old seedlings from *JcHDZ07* transgenic and wild-type plants subjected to salinity stress (150 mM NaCl) for 4 or 5 days. In vegetative growth stage, 4-week-old seedlings from *JcHDZ07* transgenic and wild-type plants subjected to salinity stress (150 mM NaCl) for 4 days.

### Measurements of Physiological Parameters

For REL measurements, about 0.2 g leaf samples were washed five times with deionized water, then placed in test tubes, followed by 10 mL of deionized water. Each sample was vibrated continuously at 25°C for 2 h, then the conductivity (B1) of the solution was measured using a top conductivity meter. Next, each sample was boiled for 25 min, the resulting solution was cooled to room temperature, the conductivity (B2) was measured again and REL was simply calculated from REL (%) = B1/B2 × 100. Previously described methods were used to measure samples’ MDA contents (Xia et al., 2018), proline contents (Bates et al., 1973), and activities of SOD and CAT in leaves from wild-type and transgenic *Arabidopsis* (Tang et al., 2017). For MDA, leaf samples from unstressed and stressed wild-type and transgenic plants were ground in 5% (w/v) trichloroacetic acid (TCA) and reacted in 0.67% (w/v) thiobarbituric acid (TBA) for 0.5 h. After cooling and centrifuge, absorbance of the resulting supernatant was measured at the wave length of 532, 600, and 450 nm, respectively. The MDA content was calculated based on the following equation: 6.45 × (OD_532_ - OD_600_) - 0.559 × OD_450_.

For proline content, about 0.5 g of *Arabidopsis* leaves were ground into powder with liquid nitrogen and extracted in 3% sulfosalicylic acid. After centrifuging at 12,000 g for 10 min, the supernatant (2 mL) was mixed with 2 mL of ninhydrin reagent [2.5% (w/v) ninhydrin, 60% (v/v) glacial acetic acid, and 40% 6 M phosphoric acid] and 2 mL of glacial acetic acid, incubated at 100°C for 40 min. Then, the reaction was terminated in an ice bath. The reaction mixture was extracted with 4 mL of toluene and the absorbance was measured at 520 nm with a UV-5200 spectrophotometer.

For CAT activity, total protein from *Arabidopsis* leaves was extracted with 0.05 M potassium phosphate buffer (pH 7.0). After centrifuging at 12,000 g for 15 min at 4°C, the supernatant was used for the measurement of CAT activity. The 5 mL reaction mixture contained 0.1 mL of the supernatant, 2.9 mL of 0.05 M potassium phosphate buffer (pH 5.5), 1 mL of 0.5% (v/v) H_2_O_2_, and 1 mL of 0.05 M guaiacol as substrates. The oxidation of guaiacol was monitored by the absorbance measured at 470 nm every 10 s. Catalase activity was confirmed using a Catalase Assay Kit (Beyotime) according to the manufacturer’s instructions.

For SOD activity, 3 mL of the mixture contained 13 mM methionine, 0.025 mM nitroblue tetrazolium (NBT), 0.1 mM EDTA, 50 mM phosphate buffer (pH 7.8), 50 mM sodium bicarbonate, and 0.5 mL enzyme extract. The reaction was started by adding 0.002 mM riboflavin and the tubes were shaken and placed under two 15-W fluorescent lamps. Illumination was started to initiate the reaction at 30°C. The reaction was allowed to proceed for 15 min, stopped by switched off the lights and covering the tubes with black cloth. The reaction medium without enzyme developed maximal color, while the non-irradiated reaction mixture served as blanks. Absorbance was measured at 560 nm.

### RNA Isolated and qRT-PCR Analysis

Total RNA was extracted from different organs of physic nut and *Arabidopsis* that had been sampled and stored at -80°C, using a MiniBEST plant RNA extraction kit (TaKaRa Code No. 9769). 2 μg RNA samples were used to synthesize first-strand cDNA using M-MLV reverse transcriptase (Promega)^3^ following the manufacturer’s instructions.

Quantitative real-time PCR (qRT-PCR) was performed using the LightCycler 480 Real-Time system (Roche, United States) in a reaction volume of 20 μL containing 10 μL of 7.2 μL of ddH_2_O, 2 μL of cDNA, 2 × SYBR Premix ExTaq, 0.4 μL forward primer (10 μM), and 0.4 μL reverse primer (10 μM). The qRT-PCR reaction conditions used in this study were as follows: 95°C for 2 min, followed by 40 cycles of 95°C for 15 s, 60°C for 1 min. The primers employed were listed in Supplementary Table S1. Relative transcriptional abundance was calculated using the 2^-ΔΔCT^ method, and *JcActin* and *AtActin2* were used as internal reference genes for physic nut and *Arabidopsis*, respectively. Three biological replicates and two technical replicates of each biological replicate were used in this experiment.

### Statistical Analysis

The data in this research of salinity stress tolerance assays, physiological index and qRT-PCR were subjected to statistical analysis using the SAS software package by Duncan’s multiple range test (Duncan, 1955).

## Results

### *JcHDZ07* Encodes a HD-Zip Protein That Belongs to the HD-Zip I Subfamily

The full-length cDNA sequence of a HD-Zip transcription factor, *JcHDZ07* (GenBank Accession JCGZ_09617) was isolated from physic nut total RNA extracted from leaves and roots by RT-PCR. Sequence analysis suggested that *JcHDZ07* contained 1566 nucleotides with a 837 bp ORF. It encoded a putative polypeptide of 278 amino acids with a predicted molecular mass of 31.6 kD and a pI of 4.71.

**FIGURE 1:**
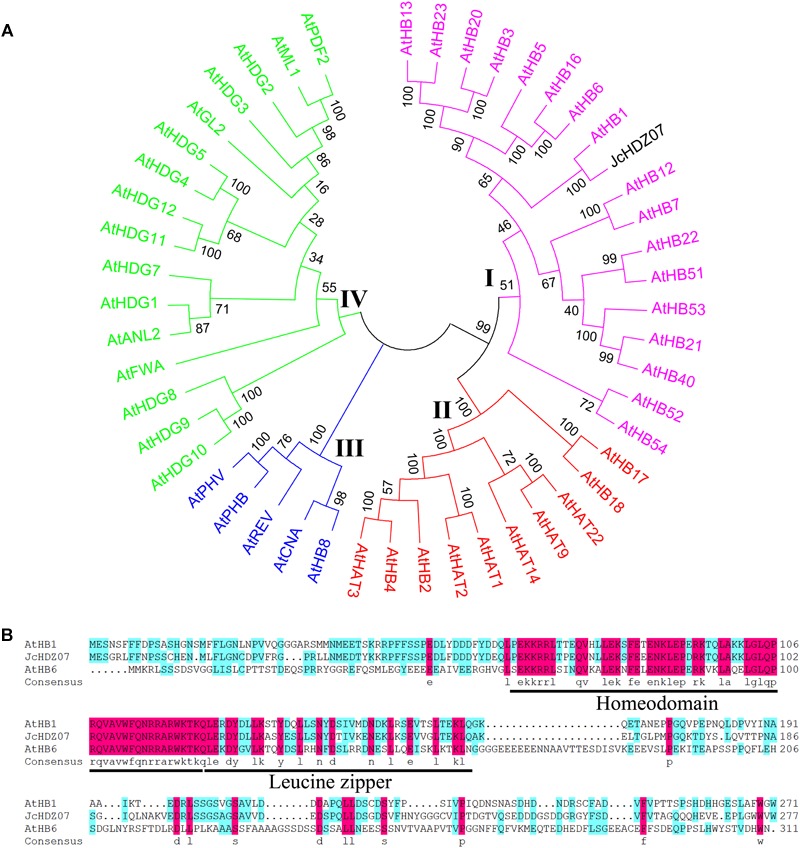
Bioinformatics analysis of *JcHDZ07*. **(A)** Phylogenetic tree analysis of JcHDZ07 and HD-Zip proteins from *Arabidopsis*. The unrooted tree was constructed, using the MEGA6.0 program, by the neighbor-joining method. **(B)** Sequence alignment of the deduced JcHDZ07 protein with known homologs. The comparison was conducted by DNAMAN (version 6.0).

We also performed a phylogenetic analysis to investigate the evolutionary relationships among JcHDZ07 and *Arabidopsis* HD-Zip proteins. Our result indicated that the HD-Zip family proteins of *Arabidopsis* and physic nut could be divided into four groups (Figure 1A), designated I–IV based on the previous classification of members in *Arabidopsis* (Ariel et al., 2007), and JcHDZ07 protein belonged to the HD-Zip I subfamily. In addition, amino acid analysis revealed that the JcHDZ07 was a HD-Zip protein with one conserved homeodomain and leucine zipper domain (Figure 1B).

### Expression Patterns of *JcHDZ07*

**FIGURE 2:**
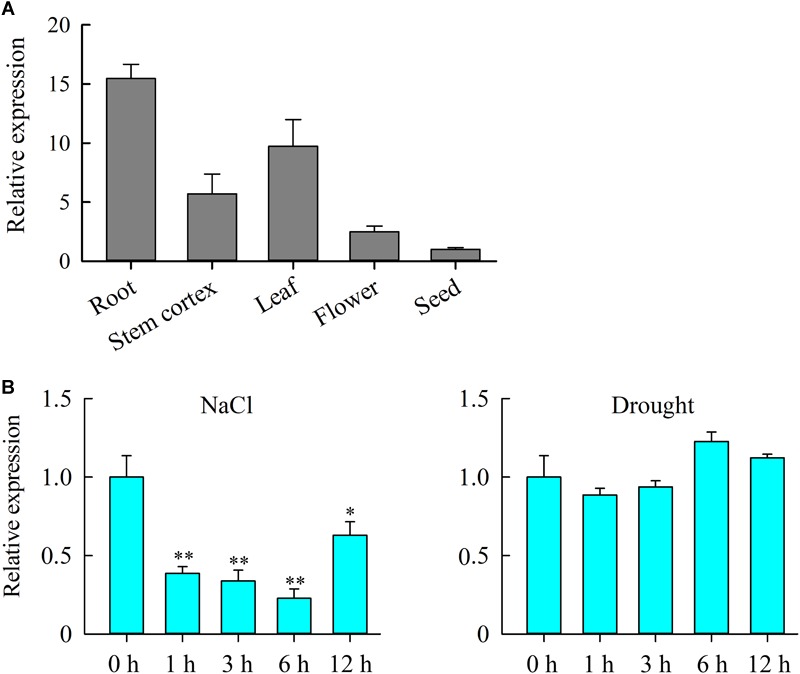
Expression profiles of *JcHDZ07* gene. **(A)** Detection of *JcHDZ07* expression in various tissues and organs using qPCR. The 2^-ΔΔCT^ method was used in qRT-PCR analysis. Relative expression levels of *JcHDZ07* were normalized by the transcript level of the *JcActin* gene and the expression level was set as 1 in seed. Values are means ± SD of three replicates. **(B)** Expression levels of *JcHDZ07* under various abiotic stresses. Zero represents leaf sample without any treatment; 1, 3, 6, and 12 represent samples after 1, 3, 6, and 12 h treatment, respectively. The 2^-ΔΔCT^ method was used in qRT-PCR analysis. Relative expression levels of *JcHDZ07* were normalized by the transcript level of the *JcActin* gene and the expression level was set as 1 at 0 h after treatment. Values are means ± SD of three replicates. Asterisks above the bars indicate the significant differences from controls at *p* < 0.01 based on three biological replicates.

To gain more insight into the role of *JcHDZ07* gene in plant growth and development, we examined the expression of *JcHDZ07* gene in roots, stem cortexes, leaves, flowers, and seeds by qRT-PCR (Figure 2A). Our results indicated that *JcHDZ07* gene was expressed in all tested tissues. In addition, the expression of *JcHDZ07* gene was highest in roots, followed by leaves, but low expression level in seeds.

We further examined the levels of expression of *JcHDZ07* gene under drought and salinity stress conditions in physic nut leaves (Figure 2B). For salt stress, the expression of *JcHDZ07* was significantly and quickly suppressed, and showed a continuous decline after 1–6 h following the treatment. Under drought stress, the transcripts of *JcHDZ07* began to decline slightly after 3 h drought treatment and gradually rise after 6 h drought stress. However, no obvious difference in the transcript of the *JcHDZ07* gene was observed after 1, 3, 6, and 12 h of drought treatment as compared with that of non-treated controls. Collectively, these results show that *JcHDZ07* is suppressed under salinity stimulation, which displays that it functions during salinity stress.

### JcHDZ07 Is a Nuclear-Localized Protein

**FIGURE 3:**
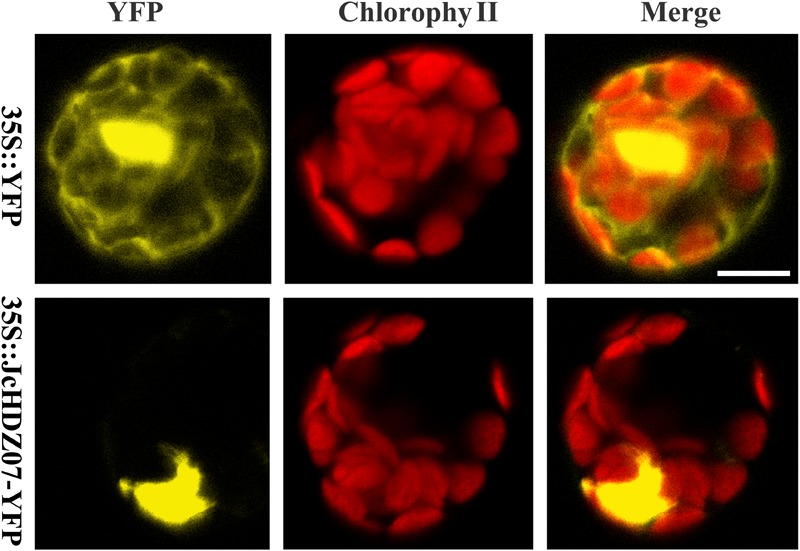
Subcellular localization of *JcHDZ07* gene in *Arabidopsis* protoplasts incubated with 35S::YFP or 35S::JcHDZ07-YFP constructs, as described in section “Materials and Methods.” Bar = 10 μm.

To determine the subcellular localization of JcHDZ07 protein, the full-length coding sequence of *JcHDZ07* without stop codon was cloned and the expression vector for JcHDZ07-YFP (yellow fluorescent protein) fusion protein was constructed. The fusion vector of the JcHDZ07-YFP fusion gene (35S::JcHDZ07-YFP) and YFP alone (35S::YFP) were transformed into *Arabidopsis* protoplast cells. The results suggested that strong fluorescence signals throughout the whole cells from the control vector, but only in the nuclei of cells harboring the 35S::JcHDZ07-YFP fusion vector (Figure 3). These results suggest that *JcHDZ07* encodes a nuclear protein.

### Phenotypic Analysis of *JcHDZ07* Transgenic *Arabidopsis*

**FIGURE 4:**
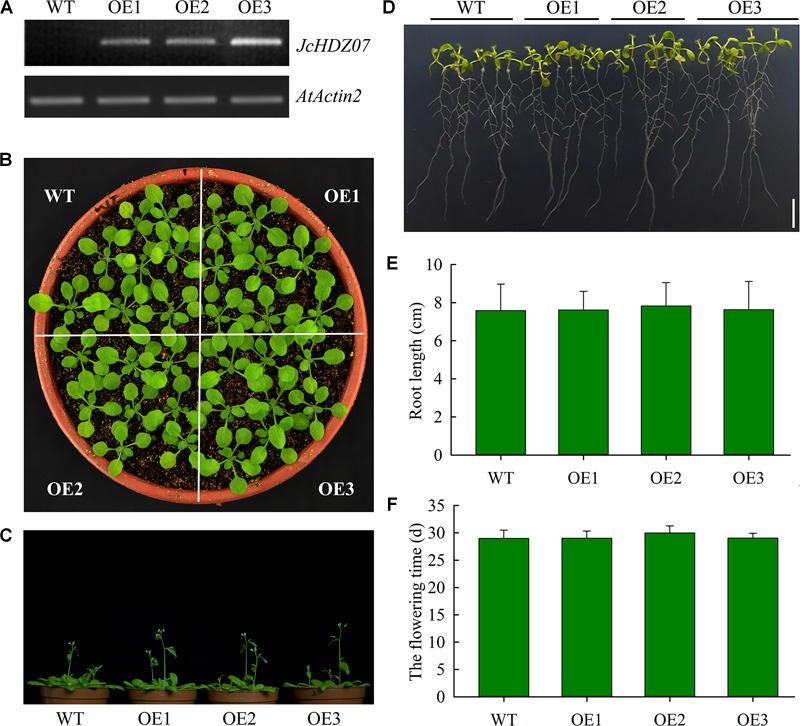
Results of phenotypic analysis in wild-type and transgenic plants. **(A)** Transcript levels of *JcHDZ07* in wild-type and transgenic *Arabidopsis*. **(B)** Phenotype of transgenic plants with *JcHDZ07* and wild-type plants. 18-day-old seedlings grown in nutrient soil and vermiculite were photographed. **(C)** Images of representative seedlings from 31-day-old wild-type and transgenic plants. **(D)** The root morphology of transgenic plants with *JcHDZ07* and wild-type plants. 11-day-old seedlings cultured in 1/2 MS were observed. **(E)** The main root lengths of 11-day-old wild-type and transgenic plants. Values represent means of *n* = 45 ± SD from three independent biological replicates. **(F)** Flowering time of the transgenic and wild-type plants: means of *n* = 45 ± SD from three independent biological replicates.

To determine the biological function of *JcHDZ07* gene, we constructed a *JcHDZ07* overexpression vector driven by the CaMV 35S promoter and introduced the vector into *Arabidopsis*. Then transgenic *Arabidopsis* plants overexpressing *JcHDZ07* were selected using hygromycin and semi-quantitative RT-PCR. RT-PCR analysis confirmed that *JcHDZ07* was expressed at the higher levels in the OE3 lines than in the OE1 and OE2 lines, but not in wild-type lines (Figure 4A). Phenotypic analysis suggested that growth of *JcHDZ07* transgenic plants was similar to that of wild-type plants (Figure 4B,C and Supplementary Figure S1A), and there were no significant differences in flowering time, plant height, 1,000-seed weight and seed yield per plant between them, under non-stressed conditions (Figure 4F and Supplementary Figures S1B–D). We further measured root length at 11 days after germination. Our results suggested that the transgenic seedlings showed no significant difference in root morphology and length than those of wild-type controls (Figure 4D,E). Thus, *JcHDZ07* expression has little apparent effect on *Arabidopsis* plants’ growth and development in the absence of stress.

### Overexpression of *JcHDZ07* in *Arabidopsis* Confers Increased Sensitivity to Salinity Stress

**FIGURE 5:**
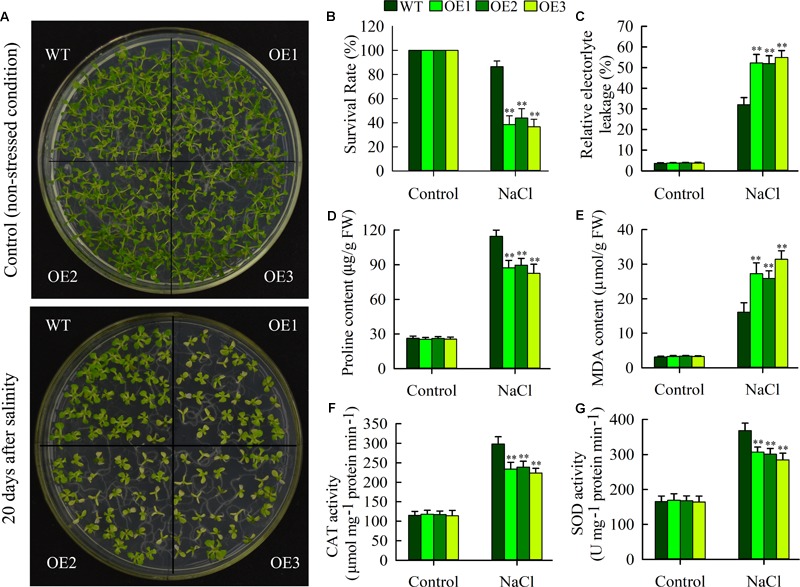
Overexpression of *JcHDZ07* in transgenic *Arabidopsis* resulted in increased sensitivity to salt stress. **(A)** Phenotypic comparison of 20-day-old seedlings of *JcHDZ07* overexpressed lines (OE1, OE2, and OE3) and WT under normal growth (non-stressed conditions) and salt stress conditions. Seeds of wild-type and transgenic plants were spotted into 1/2 MS medium containing 0 and 100 mM NaCl. **(B)** The survival rates of overexpressed lines and WT after salinity stress. **(C–G)** Relative electrolyte leakage (REL), proline contents, MDA contents and activities of catalase (CAT), and **(G)** superoxide dismutase (SOD) in leaves before and after salt treatment. Data in **(C–G)**: means of *n* = 20 ± SD from three independent experiments, asterisks above the bars indicate significant differences from wild-type controls at *p* < 0.01 according to Duncan’s multiple range test.

To examine effects of *JcHDZ07* overexpression on salinity tolerance in *Arabidopsis*, seeds of wild type and *JcHDZ07* overexpression transgenic plants (OE1, OE2, and OE3) were sowed on 1/2 MS medium containing 100 mM NaCl (for salinity stress) and 0 mM NaCl (for control) for 20 days, and then plant salinity tolerances were analyzed by comparing phenotypes. Our results indicated that leaves of most transgenic plants gradually lost greenness and their growth was severely inhibited, whereas leaves of wild-type plants remained green and their seedlings showed stronger salt stress resistance compared to transgenic plants under salt stress conditions (Figure 5A). However, we found that there was no significant difference in plant growth and development between transgenic and wild-type plants under non-stressed conditions. In addition, the survival rates of wild-type plants were significantly higher than that of transgenic plants (Figure 5B), suggesting that overexpression of *JcHDZ07* reduces the tolerance of transgenic *Arabidopsis* plants to salinity stress. We further compared the salt tolerance of the transgenic and wild-type plants at vegetative growth and adult stages. Before salinity stress treatment, the transgenic and wild-type seedlings suggested similar growth status (Supplementary Figure S2). The transgenic plant seedlings started to show leaf rolling at 4 days (Supplementary Figure S2) and became severe leaf rolling and wilting at 5 days after salinity stress (Supplementary Figure S2). However, the wild-type seedlings showed delayed and less leaf rolling and wilting symptom during the salinity stress process, as compared with the transgenic seedlings.

### Overexpression of *JcHDZ07* Does Not Affect Transgenic Plants Against Drought Stress

We further examined the resistance of transgenic plants with *JcHDZ07* to drought stress. As shown in Supplementary Figure S3, our finding suggested that there was no significant difference in the phenotype and main root length of transgenic lines with *JcHDZ07* and wild type lines under non-stressed and drought stress conditions (Supplementary Figures S3A,B,E,F). Physiological index analysis indicated that no obvious difference was observed in terms of REL and proline content under normal growth and drought stress conditions (Supplementary Figures S3C,D). In short, these results show that *JcHDZ07* overexpression plants do not alter drought stress resistance compared to wild type plants.

### Changes in Physiological Traits Under Salinity Stress Conditions

To evaluate physiological changes in transgenic plants, we further examined the REL, MDA, and proline levels as well as the activities of CAT and SOD under non-stressed and salinity stress conditions. As shown in Figure 5 and Supplementary Figures S2C,D, we found no significant difference in proline content between transgenic and wild-type lines under normal conditions, but it was clearly higher in wild-type lines under salinity stress (Figure 5D), clearly indicating that this stress response is repressed in the transgenic lines. In addition, REL and MDA levels (indicators of cell membrane damage) of transgenic plants was higher than the wild type lines under salinity stress (Figure 5C,E), indicating leaf cells are damaged less by salinity in wild-type lines than in *JcHDZ07* expressing lines. In further tests, we found that the activities of CAT and SOD were lower in transgenic leaves than in wild-type leaves under salinity stress, but not under normal growth conditions (Figure 5F,G). Taken together, these data strongly confirm that transgenic expression of *JcHDZ07* can increase the sensitivity of transgenic *Arabidopsis* to salinity stress.

### Transcript Analysis of Salinity Stress-Responsive Genes

**FIGURE 6:**
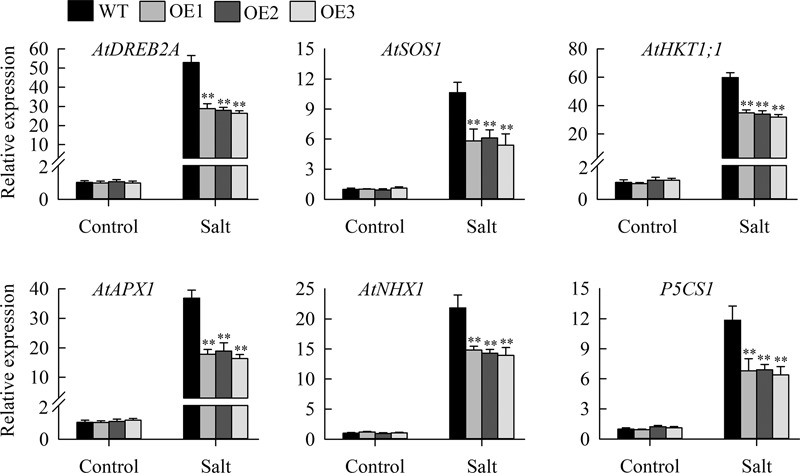
Relative expression levels of salt stress-responsive genes, in an experiment with three biological replicates, each with two technical replicates (means of *n* = 6 ± SD, asterisks above the bars indicate significant differences from wild-type controls at *p* < 0.01). The 2^-ΔΔCT^ method was used in qRT-PCR analysis. Relative expression levels were normalized by the transcript level of the *JcActin* gene as an internal control and the expression level of each gene of interest in wild-type plants under normal condition was set as 1. Values are means ± SD of three replicates.

Morphological assays suggested that transgenic lines with *JcHDZ07* had enhanced sensitivity to salinity stress. To illustrate the molecular mechanism of salt sensitivity conferred by overexpressing *JcHDZ07*, we investigated the expression of salinity-responsive genes (*AtDREB1A, AtDREB2A, AtHKT1;1, P5CS1*; *AtSOS1, AtSOS3*; *AtNHX1, AtAPX1*) in leaves of transgenic lines and wild type lines under non-stressed and salinity stress conditions using qRT-PCR. Our data suggested that their expression levels were upregulated in leaves of transgenic lines when exposed to salinity stress, but less strongly than in wild-type (Figure 6). In contrast, no clear differences in their expression levels between the wild-type and transgenic lines were detected under non-stressed conditions (Figure 6).

## Discussion

Physic nut is being widely cultivated, partly because of its rapid growth, hardiness, drought and salinity endurance, easy propagation, and adaptation to wide agro-climatic conditions (Openshaw, 2000). In *Arabidopsis* and rice, many studies have been demonstrated to elucidate the function of abiotic stress-related genes (Zhao et al., 2014; Tang et al., 2019). Results have suggested that *HD-Zip* genes are some of the most important transcription factors involved in abiotic stress signaling pathways (Song et al., 2011; Zhao et al., 2014; Bhattacharjee et al., 2016). However, no information was previously available about the molecular mechanisms involved in physic nut’s stress tolerance generally, or the functions of its *HD-Zip* genes. In our research, a HD-Zip transcription factor, *JcHDZ07* was first isolated from physic nut and then functionally characterized for its response to salinity stress resistance in transgenic *Arabidopsis*.

Numerous studies have shown that members of the HD-Zip I group are involved mainly in plant responses to abiotic stress, and overexpression of these genes can improve transgenic plants tolerance to drought and salinity (Zhang et al., 2012; Zhao et al., 2014). Similarly, our results indicated that *JcHDZ07* was assigned to group I (Figure 1), and transgenic *Arabidopsis* plants with *JcHDZ07* suggested a significant enhancement in sensitivity to salinity stress, and salinity-triggered chlorophyll loss was more pronounced in leaves of *JcHDZ07* overexpressing lines than in wild-type lines (Figure 5).

Environmental stresses (e.g., drought and salinity) often cause biochemical and physiological changes in plants, and these parameters (e.g., proline and MDA content, CAT, and SOD activity) may be used as indications for evaluating drought and salt stress resistance in crop plants (Wei et al., 2016). Proline is considered as an inert compatible osmolyte that protects macromolecules and subcellular structures when plants are exposed to drought and salinity stresses (Szabados and Savoure, 2010). Such a connection between stress tolerance and proline accumulation is supported by knocking out and overexpressing the *P5CS* gene in various plants (Chen et al., 2013). A significant lower accumulation of proline was observed in leaves of *JcHDZ07* expressing lines than in wild-type lines in response to salt stress (Figure 5D). Our results indicate that the accumulated proline may be seen as a response of transgenic plants with *JcHDZ07* toward salt stress. REL and MDA levels have been reported to be able to be used as a measure of cell membrane stability, and plants with lower REL and MDA contents often have increased resistance to abiotic stress (Tang et al., 2017). Our data indicated that salt induced lower increases in REL and MDA contents in leaves of wild-type lines than in leaves of *JcHDZ07* overexpressing lines (Figure 5C,E). These finding indicate that salt causes more cell membrane damage in leaves of *JcHDZ07* overexpressing plants, corroborating *JcHDZ07*’s role in negative regulation of salt responses. Salinity impose osmotic stress, which leads to the production of antioxidant enzymes such as CAT and SOD, and the up-regulation of the activities of CAT and SOD protects plants against oxidative damage under salt stress conditions (Wei et al., 2016). In our study, transgenic *Arabidopsis* expressing *JcHDZ07* suggested lower CAT and SOD activities than wild type plants following salinity stress (Figure 5F,G), indicating that salinity may induce more severe oxidative damage in them than in wild-type plants. Thus, these results at least partially explain the increased sensitivity to salinity of our transgenic rice plants.

The increased sensitivity to salt stress also attributable to obviously reduced transcript of stress-related genes (Bhattacharjee et al., 2016; Tang et al., 2017). For example, overexpression of *OsHOX24* enhances the sensitivity of transgenic *Arabidopsis* to salinity stress by regulating the transcript of abiotic stress-related genes (Bhattacharjee et al., 2016). Similarly, our findings suggested that several stress responsive genes (*AtDREB1A, AtDREB2A, AtHKT1;1, P5CS1*; *AtSOS1, AtSOS3*; *AtNHX1, AtAPX1*) were up-regulated in the *JcHDZ07*-overexpressing *Arabidopsis* plants under salinity stress conditions, but less strongly compared with wild-type plants (Figure 6). The *Arabidopsis SOS1* gene encodes a plasma membrane Na^+^/H^+^ antiporter, and transgenic plants overexpressing *SOS1* increase tolerance to salt stress (Shi et al., 2003). In addition, the constitutive expression of *AtSOS3* has been shown to improve the salinity tolerance of *Arabidopsis* (Yang et al., 2009). *Arabidopsis* Na^+^/H^+^ antiporter *AtNHX1* is reported to confer salt tolerance on transgenic plants (Apse et al., 1999). Overexpression of *AtDREB1A* and *AtDREB2A* confers lower sensitivity to salt stress in transgenic seedlings (Hong et al., 2006; Hussain et al., 2018). *AtHKT1;1* gene products are vital for salt resistance in *Arabidopsis* by venting sodium ions from sensitive cells, and *AtHKT1;1* overexpressing plants reduce salt sensitivity in transgenic *Arabidopsis* (Møller et al., 2009). The products of the *AtAPX1* gene are involved in the scavenging of reactive oxygen species (Fourcroy et al., 2004). In short, our results strongly show that *JcHDZ07* negatively regulates salinity responses in our transgenic *Arabidopsis* lines at least partly through down-regulation of known salt stress-related genes. Taken together, we summarized a simple working model of *JcHDZ07*. When plants suffer from salt stress, which alters the expression of *JcHDZ07* and other salinity-responsive genes. Then, the *JcHDZ07* protein starts or inhibits the expression of salt-responsive genes, which makes the plants more sensitive to salt stress.

## Conclusion

In conclusion, we report the physic nut HD-Zip I subfamily gene *JcHDZ07*, which is inhibited by salinity. Ectopic overexpression of *JcHDZ07* in *Arabidopsis* confers reduced the tolerance of *Arabidopsis* plants to salt stress through the regulation of the changes in physiological indexes and transcript of stress responsive genes. These results will provide a basis for further study the biological functions of the *HD-Zip* genes in physic nut. In the future, more efforts may be required to elucidate the detailed regulatory mechanisms underlying *JcHDZ07*’s salinity stress sensitivity in physic nut.

## Data Availability

All datasets generated for this study are included in the manuscript and/or the Supplementary Files.

## Author Contributions

YT conceived and designed the experiments. SW, YL, JT, RD, and MY performed the experiments. XY and MZ analyzed the data. YL and XB wrote and revised the manuscript. All authors read and approved the final manuscript.

## Conflict of Interest Statement

The authors declare that the research was conducted in the absence of any commercial or financial relationships that could be construed as a potential conflict of interest.

## Footnotes

1 https://www.arabidopsis.org/

2 https://www.ncbi.nlm.nih.gov/

3 http://www.promega.com

## Supplementary Material

FIGURE S1 Results of phenotypic analysis in wild-type and transgenic (OE1, OE2, and OE3) *Arabidopsis* plants expressing *JcHDZ07*. **(A)** Images of representative seedlings. **(B)** Plant height of the transgenic and wild-type plants: means of *n* = 45 ± SD from three independent biological replicates. **(C)** The 1000-seed weight. Seed weights were calculated by randomly selected seeds. The mature seeds were dried under 37°C in an oven for 3 days. Values represent means of *n* = 45 ± SD from three independent experiments. **(D)** Seed yield of the wild-type and transgenic plants under normal growth conditions. Values represent means of *n* = 45 ± SD from three independent experiments.

FIGURE S2 Increased salinity sensitivity in transgenic plants with *JcHDZ07* gene. **(A)** Phenotype of the transgenic and wild-type plants at different stages during the salinity stress experiments (150 mM NaCl). 10-week-old seedlings from *JcHDZ07* transgenic and wild-type plants subjected to salinity stress for 4 or 5 days. **(B)** Phenotype of the transgenic and wild-type plants at different stages during the salinity stress experiments. 4-week-old seedlings from *JcHDZ07* transgenic and wild-type plants subjected to salinity stress for 4 days. **(C,D)** Relative electrolyte leakage (REL) **(C)** and proline content **(D)** in leaves of 10-week-old seedlings before salt stress and 3 days after salt treatment. Data in **(C,D)**: means of *n* = 20 ± SD from three independent experiments, asterisks above the bars indicate significant differences from wild-type controls at *p* < 0.01 according to Duncan’s multiple range test.

FIGURE S3 Overexpression of *JcHDZ07* in transgenic *Arabidopsis* did not alter transgenic plant’s tolerance to drought stress. **(A)** Phenotypic comparison of *JcHDZ07* overexpressed lines (OE1, OE2, and OE3) and WT under normal growth and drought stress conditions. **(B)** The length of main roots from 11-day-old wild-type and transgenic plants under non-stressed and drought stress conditions. **(C)** Relative electrolyte leakage in leaves before and after drought treatment. **(D)** Proline content in leaves before and after drought treatment. Data in **(B–D)**: means of *n* = 20 ± SD from three independent experiments. **(E)** Performance of 4-week-old seedlings from *JcHDZ07* transgenic and wild-type plants subjected to drought stress without water for 26 days and **(F)** then recovered for 6 days.

TABLE S1 Primers used in this study.
