# Supplementary figures and images for: A Physic Nut Stress-Responsive HD-Zip Transcription Factor, JcHDZ07, Confers Enhanced Sensitivity to Salinity Stress in Transgenic Arabidopsis
Source: Front Plant Sci. 2019 Jul 17;10:942. doi: 10.3389/fpls.2019.00942 (PMC6652468; doi:10.3389/fpls.2019.00942)

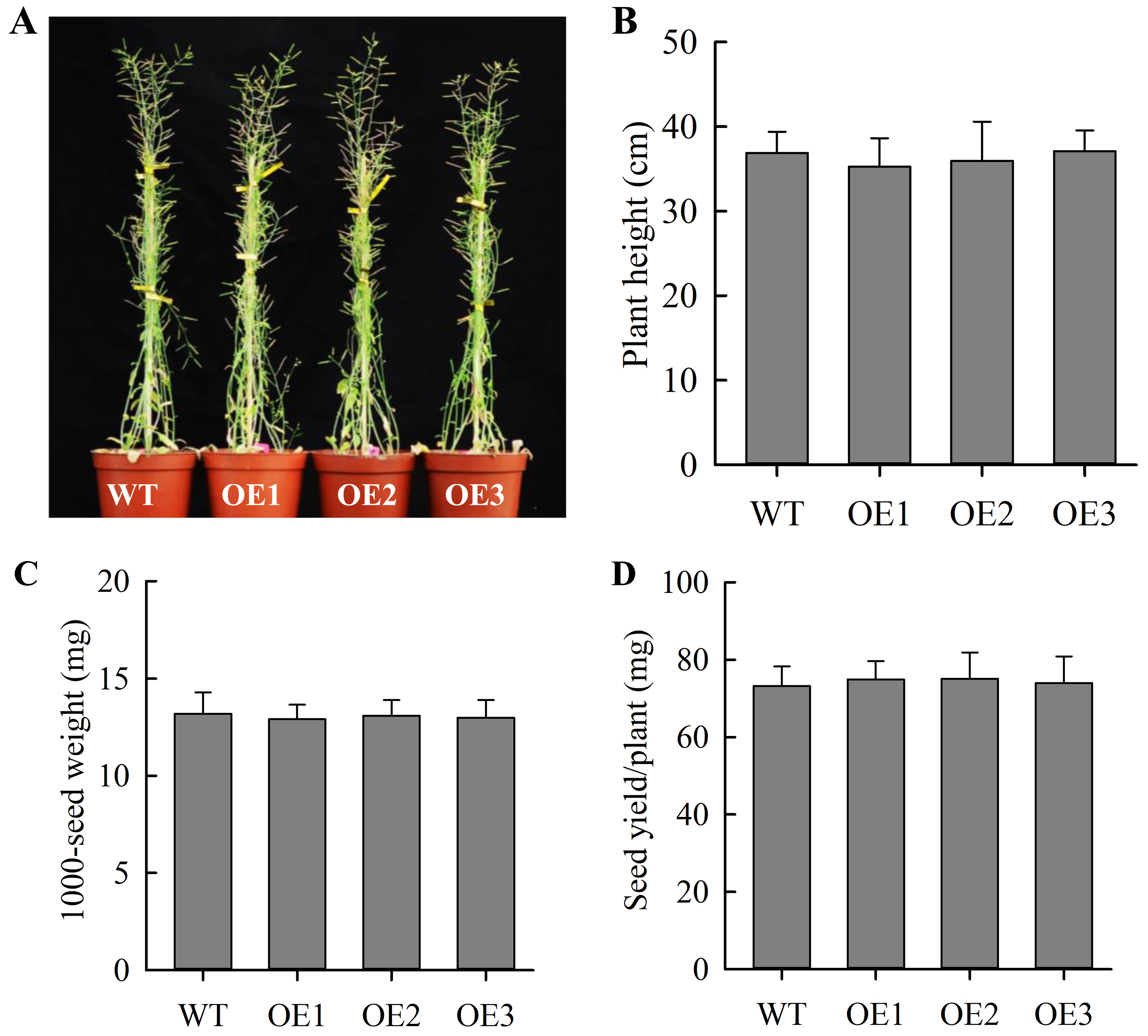

Supplement: FIGURE S1 — Results of phenotypic analysis in wild-type and transgenic (OE1, OE2, and OE3) Arabidopsis plants expressing JcHDZ07. (A) Images of representative seedlings. (B) Plant height of the transgenic and wild-type plants: means of n = 45 ± SD from three independent biological replicates. (C) The 1000-seed weight. Seed weights were calculated by randomly selected seeds. The mature seeds were dried under 37°C in an oven for 3 days. Values represent means of n = 45 ± SD from three independent experiments. (D) Seed yield of the wild-type and transgenic plants under normal growth conditions. Values represent means of n = 45 ± SD from three independent experiments. [file Image_1.TIF]

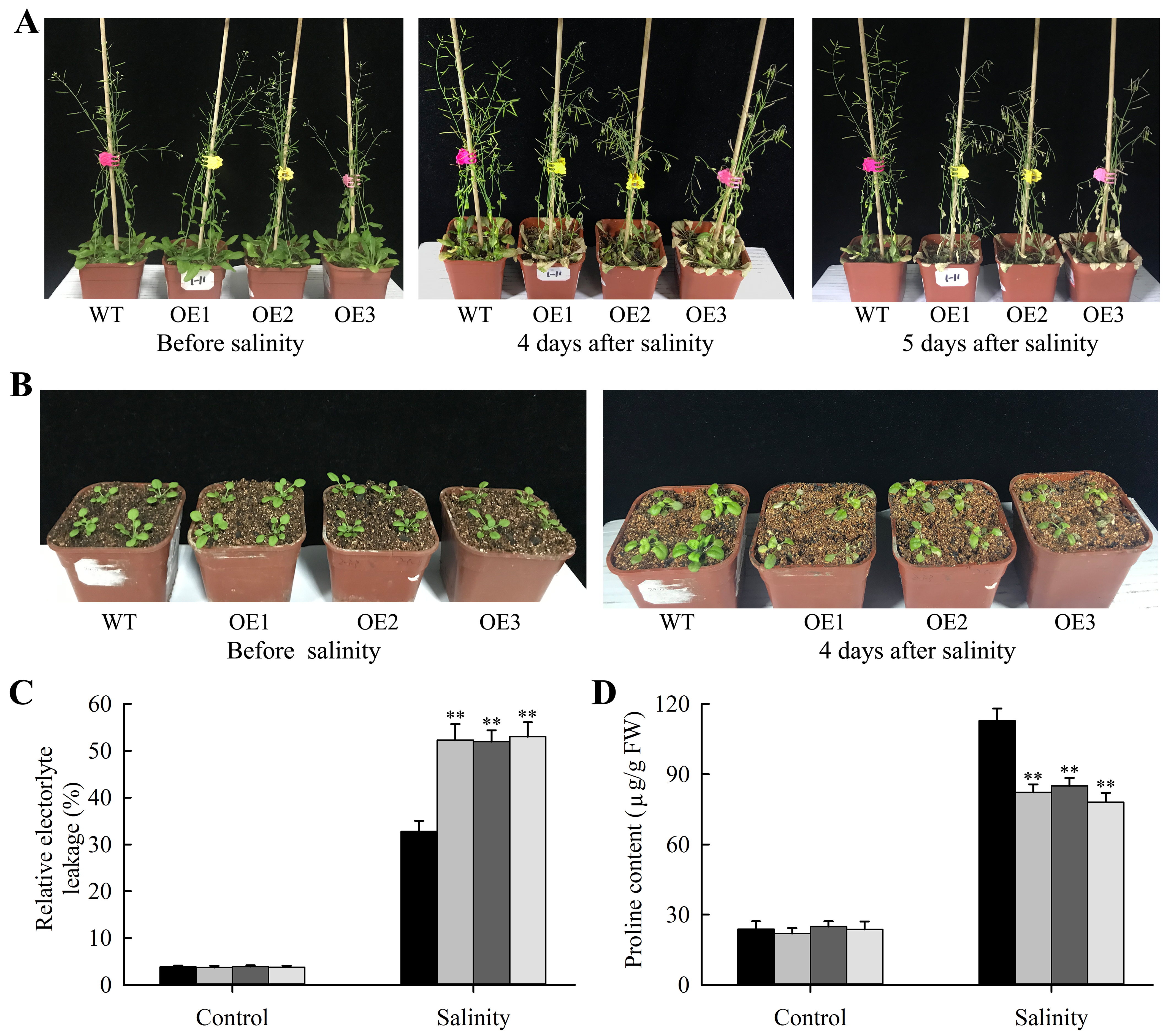

Supplement: FIGURE S2 — Increased salinity sensitivity in transgenic plants with JcHDZ07 gene. (A) Phenotype of the transgenic and wild-type plants at different stages during the salinity stress experiments (150 mM NaCl). 10-week-old seedlings from JcHDZ07 transgenic and wild-type plants subjected to salinity stress for 4 or 5 days. (B) Phenotype of the transgenic and wild-type plants at different stages during the salinity stress experiments. 4-week-old seedlings from JcHDZ07 transgenic and wild-type plants subjected to salinity stress for 4 days. (C,D) Relative electrolyte leakage (REL) (C) and proline content (D) in leaves of 10-week-old seedlings before salt stress and 3 days after salt treatment. Data in (C,D): means of n = 20 ± SD from three independent experiments, asterisks above the bars indicate significant differences from wild-type controls at p < 0.01 according to Duncan’s multiple range test. [file Image_2.TIF]

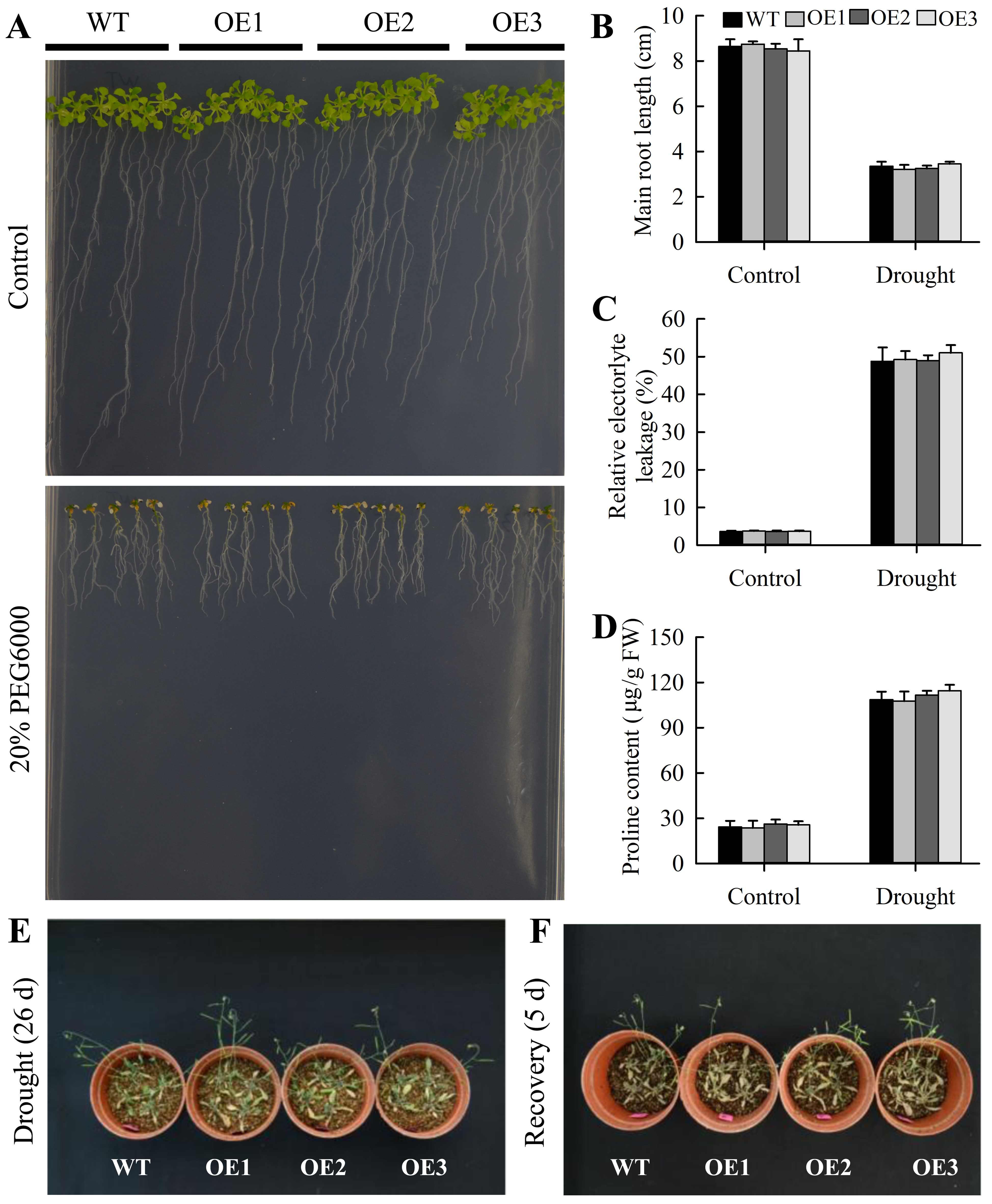

Supplement: FIGURE S3 — Overexpression of JcHDZ07 in transgenic Arabidopsis did not alter transgenic plant’s tolerance to drought stress. (A) Phenotypic comparison of JcHDZ07 overexpressed lines (OE1, OE2, and OE3) and WT under normal growth and drought stress conditions. (B) The length of main roots from 11-day-old wild-type and transgenic plants under non-stressed and drought stress conditions. (C) Relative electrolyte leakage in leaves before and after drought treatment. (D) Proline content in leaves before and after drought treatment. Data in (B–D): means of n = 20 ± SD from three independent experiments. (E) Performance of 4-week-old seedlings from JcHDZ07 transgenic and wild-type plants subjected to drought stress without water for 26 days and (F) then recovered for 6 days. [file Image_3.TIF]
